# In Vitro Antiparasitic Effects of Silver Nanoparticles on *Sarcocystis* Bradyzoites

**DOI:** 10.1002/vms3.71092

**Published:** 2026-07-15

**Authors:** Nasrin Karimi, Mir‐Hassan Moosavy, Nasser Hajipour, Hamed Behniafar

**Affiliations:** ^1^ Department of Food Hygiene and Aquatics Faculty of Veterinary Medicine University of Tabriz Tabriz Iran; ^2^ Department of Medical Parasitology Sarab Faculty of Medical Sciences Sarab East Azerbaijan Iran

**Keywords:** antiparasitic activity, in vitro, *Sarcocystis*, silver nanoparticles

## Abstract

**Background:**

*Sarcocystis* is a protozoan parasite of significant zoonotic and economic importance, primarily transmitted through the consumption of contaminated meat. Conventional antiparasitic drugs show limited efficacy and potential side effects, leading to growing interest in nanotechnology‐based alternatives.

**Objective:**

This study evaluated the in vitro antiparasitic activity of silver nanoparticles (AgNPs) against *Sarcocystis* bradyzoites isolated from sheep.

**Methods:**

Macroscopic cysts were collected from infected sheep muscles at the Tabriz abattoir, and bradyzoites were purified and confirmed to be viable using trypan blue staining and a bioassay in cats. The parasites were exposed to AgNP concentrations of 100, 200, 400 and 800 µg/mL for 10, 30, 60 and 120 min, in triplicate. Untreated samples (phosphate‐buffered saline [PBS]) and 5% NaCl (positive control) were included for comparison. Data were analysed using one‐way ANOVA (*p* < 0.05).

**Results:**

The results demonstrated that a dose‐ and time‐dependent increase in mortality, ranging from 20.11% ± 2.49% at 100 µg/mL after 10 min to 59.53% ± 8.52% at 800 µg/mL after 120 min. Although silver nanoparticles (AgNPs) did not achieve complete inactivation, their significant effect compared to controls (*p* < 0.05) indicates moderate in vitro antiparasitic activity.

**Conclusion:**

AgNPs exhibited measurable in vitro antiparasitic activity against *Sarcocystis* bradyzoites, suggesting their promise as an adjunct or alternative to traditional drugs. Further in vivo studies are recommended to assess safety, optimal dosing and practical applications in veterinary and food safety contexts.

## Graphical abstract

In vitro, silver nanoparticles (AgNPs) exhibit dose and time‐dependent antiparasitic activity against *Sarcocystis* bradyzoites from sheep, achieving up to 59.53% mortality at 800 µg/mL after 120 min. These results suggest AgNPs may be a potential alternative to traditional drugs, meriting further in vivo investigation for veterinary use.

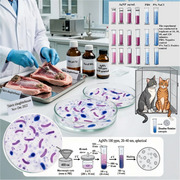

## 1 Introduction

Sarcocystosis is a protozoan parasitic disease with a global distribution, caused by organisms belonging to the genus *Sarcocystis*. The genus belongs to the family Sarcocystidae, the order Eucoccidiorida, the subclass Coccidiasina, the class Sporozoasida and the phylum Apicomplexa. Members of this genus can infect various warm‐ and cold‐blooded vertebrates, including birds, mammals and reptiles (Decker Franco et al. 2017). Species within this genus of protozoa have a life cycle that is obligately heteroxenous; sexual reproduction and subsequent oocyst formation take place in the intestinal mucosa of the definitive host, whereas asexual reproduction, comprising the schizont and sarcocyst stages, occurs within the endothelial and smooth muscle cells of the intermediate host, respectively (Dubey and Sykes 2021).

So far, more than 200 species of *Sarcocystis* have been identified, which can infect humans and various animals, including domestic animals such as cattle and sheep, thereby harming human society (Dubey and Sykes 2021). Zoophilic species are capable of causing diseases that lead to clinical signs, including loss of appetite, weight loss, fever, anaemia, muscle weakness, reduced milk and meat production, abortions and sometimes even the death of their intermediate hosts, such as cattle, sheep and goats (Dubey and Rosenthal 2023; Feng et al. 2023). Moreover, humans are the definitive hosts for two species, *Sarcocystis hominis* and *Sarcocystis suihominis*, which are transmitted through raw or undercooked contaminated meat. These species can lead to various symptoms, including abdominal pain, diarrhoea, vomiting and flatulence (Fayer et al. 2015).

Despite the importance of this parasite and the problems it causes for public health and food security, there is currently no well‐established specific treatment available (Fayer et al. 2015). As infections are often self‐limiting, the treatments used in humans and animals are generally supportive and symptomatic (Latif and Muslim 2016). Consequently, the combination of anthelmintic drugs such as albendazole and maintenance treatments, such as the use of corticosteroids, is currently prescribed (Fayer et al. 2015).

However, these conventional treatments have limited efficacy and may be associated with undesirable side effects or drug resistance. Therefore, alternative control measures such as proper cooking, freezing or heating of meat to inactivate bradyzoites in infected muscle tissues have gained attention in recent years (Valizadeh 2021). Nevertheless, such physical methods are only effective in preventing transmission via meat and cannot address systemic infections or the persistence of parasites in the host.

In light of these limitations, new and more effective strategies are needed to control *Sarcocystis* infections. One promising approach is the use of nanotechnology, which has recently attracted increasing attention in parasitology and antimicrobial research.

Nanoparticles can improve drug delivery and cellular uptake, and they generate reactive oxygen species (ROS) that damage microbial cells (Mercan et al. 2022; Solanki et al. 2024). Their small size allows close interaction with the surface of pathogens, enhancing their antimicrobial effects (Draviana et al. 2023). Among these, silver nanoparticles (AgNPs) have shown strong activity against several protozoan infections, including *Toxoplasma gondii*, *Leishmania*, *Cryptosporidium* and *Blastocystis hominis* (Cameron et al. 2016; Karimipour et al. 2024; Majeed et al. 2024; Younis et al. 2020).

AgNPs affect pathogens through several mechanisms, most notably by generating ROS, disrupting microbial membranes, and interfering with DNA and protein functions (Ali et al. 2023; Mikhailova 2024). These properties make AgNPs promising agents as alternative or adjunctive therapies to conventional drugs, especially given the increasing concerns regarding drug resistance in protozoan diseases. Additionally, the nanoscale size of AgNPs enhances their cellular uptake and bioavailability, enabling more targeted antimicrobial effects with possibly reduced host toxicity.

To the best of our knowledge, the efficacy of silver nanoparticles (AgNPs) against members of the genus *Sarcocystis* has not yet been evaluated. Therefore, the present study was designed to investigate the in vitro efficacy of AgNPs in killing *Sarcocystis* bradyzoites isolated from sheep.

## 2 Materials and Methods

### 2.1 Sampling

Sampling was performed during November and December 2022. *Sarcocystis* specimens were obtained from the diaphragm, intercostal and masseter muscles of sheep slaughtered at the Tabriz slaughterhouse. Macroscopic signs of contamination were evident in the samples, which were then transported under cold chain conditions to the Food and Aquatic Hygiene Laboratory.

All sampling procedures were conducted under aseptic conditions to prevent cross‐contamination, and each specimen was processed within 4 h post‐slaughter to preserve parasite viability.

### 2.2 Microscopic Examination

Microscopic techniques were utilized to verify the presence of the parasite in the obtained samples. This approach is commonly employed to confirm *Sarcocystis* infection by observing characteristic morphological features under light microscopy.

### 2.3 Impression Smear

Approximately 1 g of the collected muscle tissue was sliced into small pieces about 3–5 mm thick, then thoroughly crushed between two glass slides. Following Giemsa staining, the samples were observed under a microscope at 400× magnification. The presence of banana‐shaped bradyzoites was used as a diagnostic criterion for confirmation of infection.

### 2.4 Purification of Bradyzoites

One hundred macroscopic cysts were dissolved in phosphate‐buffered saline (PBS) at pH 7.0, passed through a 40‐mesh sieve, and centrifuged at 1000 × *g* for 10 min. PBS was added to the resulting pellet, followed by layering with 90% Percoll solution containing 9% sodium chloride to form a gradient. This suspension was centrifuged at 100 × *g* for 10 min, and the pellet obtained was washed three times with PBS to purify the bradyzoites. Finally, bradyzoite viability was assessed using 4% trypan blue staining (Honda et al. 2018). All centrifugation steps were carried out at 4°C to maintain cellular integrity. Bradyzoite purity was confirmed microscopically before subsequent treatments.

### 2.5 Evaluation of Bradyzoite Viability (Bioassay)

Several cysts were fed to two cats to evaluate the viability or mortality of isolated bradyzoites before treatment with AgNPs. Between Days 10 and 12 post‐feeding, faecal samples from the cats were examined for parasite oocysts using the Sheather flotation method (Verma et al. 2017). Before cyst administration, the cats were treated with clindamycin at 12 mg/kg for 2 weeks to ensure clearance of any existing parasites.

### 2.6 Silver Nanoparticles

Commercial silver nanoparticle solution at 1000 ppm (equivalent to 1 g/L) was obtained from Pishgaman Nano Materials Iranian Company. Various 100, 200, 400 and 800 µg/mL concentrations were prepared using the dilution formula C1V1 = C2V2. Moreover, 5% NaCl was used as a positive control, whereas PBS served as the negative/control treatment. Sodium chloride is known to reduce the viability of *Sarcocystis* bradyzoites, making it an effective positive control for inactivation studies (Honda et al. 2018). According to the manufacturer, the AgNPs had an average particle size of 20–40 nm and a spherical morphology.

### 2.7 Treatment of Sarcocyst Bradyzoites Using AgNPs

Following confirmation of bradyzoite viability, several cysts were homogenized in PBS at pH 7.0 and treated with various concentrations of silver nanoparticles for 10, 30, 60 and 120 min, each treatment repeated in triplicate. The viability of bradyzoites post‐treatment was then assessed using the previously described method. All treatments were performed at room temperature (approximately 25°C) with gentle agitation to ensure uniform exposure.

### 2.8 Statistical Analysis

Version 21 of the SPSS software was used to analyse the results obtained. To evaluate statistical significance, Duncan's and one‐way ANOVA tests were employed with a 95% confidence interval. A *p* value of <0.05 was considered statistically significant, and results were presented as mean ± standard deviation.

## 3 Results

**FIGURE 1:**
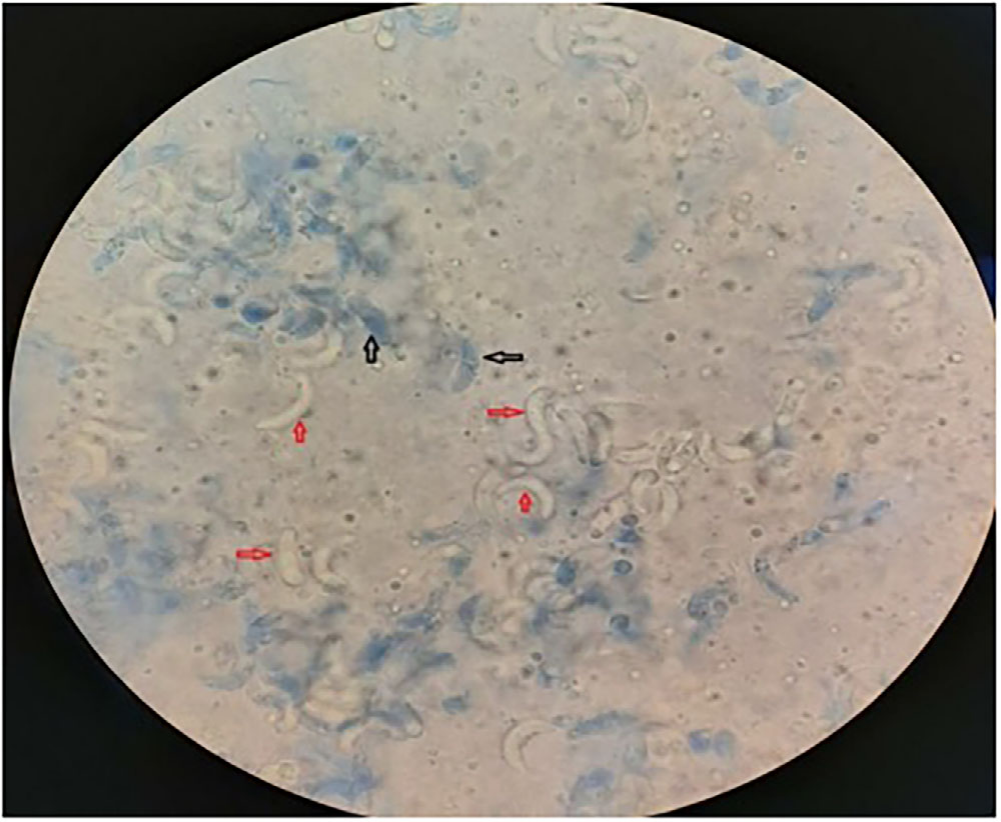
Viable (indicated by red arrows) and non‐viable (indicated by black arrows) *Sarcocystis* bradyzoites were identified after staining with trypan blue under 100× magnification.

Using trypan blue staining and microscopic observation, the viability of bradyzoites was evaluated (Figure 1). In this method, live bradyzoites exclude the dye and remain unstained, whereas dead bradyzoites take up the trypan blue and appear stained under the microscope.

The results demonstrated that the lowest effectiveness of AgNPs was at a concentration of 100 µg/mL after 10 min, with an efficacy of 20.11%. In contrast, the highest effectiveness was observed at 800 µg/mL after 120 min, reaching 59.53% (Table 1). The positive control (5% NaCl) achieved 100% mortality at 120 min, whereas the negative control (PBS) maintained more than 85% viability, confirming the assay's reliability.

**TABLE 1:** Mortality rates of *Sarcocystis* bradyzoites (mean ± standard deviation) at various concentrations (µg/mL) of silver nanoparticles and different exposure times (minute).

Time concentration	10	30	60	120
100	20.11 ± 2.49^c^	25.13 ± 4.32^cd^	27.22 ± 4.97^bc^	27.86 ± 2.62^bc^
200	29.91 ± 2.83^bc^	28.86 ± 5.35^cd^	33.01 ± 5.35^bc^	30.01 ± 5.35^bc^
400	30.83 ± 4.24^bc^	37.78 ± 2.05^bc^	40.02 ± 1.25^b^	41.48 ± 5.66^bc^
800	44.44 ± 6.6^b^	52.60 ± 3.77^b^	40.55 ± 13.02^b^	59.53 ± 8.52^b^
Control+	81.33 ± 16.36^a^	88.89 ± 3.56^a^	95.99 ± 0.82^a^	100.00 ± 4.32^a^
Control−	8.97 ± 0.47^c^	12.81 ± 0.82^d^	13.33 ± 0.47^c^	15.21 ± 0.94^c^

*Note*: Lowercase letters (a–d) indicate statistically significant differences between mean values within each column.

## 4 Discussion

The current investigation elucidates the in vitro antiparasitic efficacy of silver nanoparticles (AgNPs) against *Sarcocystis* bradyzoites. Employing trypan blue staining for viability assessment, our data unveiled a pronounced dose‐ and time‐dependent escalation in bradyzoite mortality. Specifically, at 100 µg/mL, following 10 min of exposure, AgNPs elicited a modest 20.11% mortality, which peaked at 59.53% at 800 µg/mL after 120 min of exposure. These outcomes corroborate our central hypothesis that AgNPs harbour antiparasitic properties against *Sarcocystis*, though efficacy varied across formulations, potentially due to differences in nanoparticle synthesis or characterization. Compared to the positive control, which achieves 100% mortality by 120 min, and the negative control, exhibiting negligible effects (15.21% at 120 min), the attributable impact of AgNPs is evident. This result aligns with our objective to position AgNPs as a viable supplement or alternative to standard antiparasitics, targeting meat‐borne *Sarcocystis* transmission that inflicts substantial economic burdens on livestock industries and health risks to humans.

The highest mortality rate observed was 59.53% at a concentration of 800 µg/mL after 120 min. However, this level of inactivation is relatively modest compared to physical methods such as thorough cooking (internal temperature ≥70°C) or freezing, which achieve near‐complete inactivation of *Sarcocystis* bradyzoites. In practical meat processing, the standalone application of this efficacy may have a limited impact on reducing the transmission risk of sarcosporidiosis, particularly for human‐infecting species (*S. hominis* and *S. suihominis*). Nevertheless, partial inactivation could be a valuable component of a multi‐hurdle approach (e.g., combined with cold chain management, hygiene practices, or other antimicrobials), especially in endemic regions with varying consumer practices. In veterinary contexts, silver nanoparticles (AgNPs) might function as a supplementary tool to reduce cyst burden in live animals rather than serving as a primary meat disinfectant (Dehkordi et al. 2017; Franssen et al. 2019).

The antiparasitic mechanisms of AgNPs likely involve complex cellular interactions, drawing on antimicrobial models adapted for protozoans. AgNPs adhere to membranes, increasing permeability and promoting ion efflux, which disrupts osmotic equilibrium and precipitates lysis (Jian et al. 2022; Khalifa et al. 2025; Pimentel‐Acosta et al. 2020). They also generate ROS, which fosters oxidative stress that damages lipids, proteins and DNA, thereby impairing enzymatic activity, signal transduction pathways, and replication (Dakal et al. 2016). For example, AgNPs bind DNA, inducing conformational shifts and mutations, which may account for the time‐progressive mortality in our trials (Morones et al. 2005). The positive surface charge facilitates electrostatic adhesion to negatively charged membrane groups, such as carboxyl, phosphate and amino residues, thereby restructuring the barrier for penetration (Rashid et al. 2017). Moreover, AgNPs act as ion carriers, utilizing proton gradients to lower the local pH and accelerate silver release (Singh et al. 2016). These mechanisms collectively suggest that AgNPs can disrupt multiple vital pathways in protozoan cells rather than acting through a single target, which may explain their broad‐spectrum efficacy observed in related organisms.

Although direct mechanistic experiments on *Sarcocystis* were not conducted in this study, existing literature on related protozoan parasites indicates that silver nanoparticles (AgNPs) exert antiparasitic effects primarily through the generation of ROS, disruption of parasite membranes, interference with DNA replication and protein function, and induction of oxidative stress. These mechanisms are consistent with the observed time‐ and dose‐dependent mortality and have previously been documented against *T. gondii*, *Cryptosporidium* and *Leishmania* species. Future research should incorporate ultrastructural analyses (SEM/TEM), ROS quantification and molecular assays to verify whether similar mechanisms occur in *Sarcocystis* bradyzoites (Shojaee et al. 2019; Zhang et al. 2023).

In bacterial analogues, adhesion alters membrane traits and causes internal DNA damage (Durán et al. 2016), with the primary action being to enhance solubility and permeability (McQuillan et al. 2012). Dissolved ions interact with thiol proteins, forming complexes with electron donors involving oxygen, phosphorus, nitrogen or sulphur (Holt and Bard 2005). Reports show that AgNPs elevate the *trans*/*cis* ratio of unsaturated fatty acids, causing rigidity and bilayer fissures that impair function (Cakić et al. 2016). This disrupts biofilm initiation, which is valuable against pathogens (Mohanta et al. 2020). Silver ions target disulphide bonds in respiratory enzymes and thiols, inactivating them and amplifying ROS (Gamboa et al. 2019; Yan et al. 2018). The dip in efficacy at specific intervals, such as 40.55% at 800 µg/mL after 60 min versus 52.60% at 30 min, could stem from nanoparticle aggregation or transient adaptations of bradyzoites. However, statistical significance persisted across groups (Mikhailova 2020). While resistance emerges in some protozoans, our data suggest robustness against *Sarcocystis*.

Comparing our results with prior studies reveals both similarities and notable differences, thereby underscoring our contribution to the field. Although direct *Sarcocystis* studies are scarce, parallels with other protozoans underscore the versatility of AgNPs. For instance, AgNPs yielded over 90% inhibition against *B. hominis* at a concentration of 100 µg/mL, as determined by membrane cytotoxicity (Younis et al. 2020). Similarly, against *Entamoeba histolytica* and *Cryptosporidium parvum*, AgNPs and copper oxide nanoparticles reduced cyst viability by up to 100% at 500 µg/mL, outperforming controls through ROS‐mediated damage (Saad et al. 2015). However, the comparatively lower efficacy observed in *Sarcocystis* may be attributed to its thick cyst wall, which provides mechanical and biochemical protection against nanoparticle penetration. In contrast to *T. gondii*, where 80%–90% tachyzoite reduction occurred at 50–100 µg/mL via organelle disruption (Vergara‐Duque et al. 2020). Furthermore, another study demonstrated a 95% elimination of the protoscolex of *Echinococcus granulosus* at a concentration of 0.3 mg/mL after 120 min (Bavand et al. 2014). In addition, gold nanoparticles on *Giardia lamblia* cysts achieved 100% lethality at 0.5 mg/mL after 180 min, surpassing the efficacy of metronidazole (Bavand et al. 2014). Moreover, AgNPs have been reported to be effective in controlling and reducing secondary infection in cutaneous leishmaniasis caused by Leishmania *major* (Mohebali et al. 2015).

The practical implications of our findings are substantial, particularly in bridging research gaps related to zoonotic control. *Sarcocystis* infections lead to economic losses through reduced meat quality, abortions and decreased livestock productivity, whereas human cases cause gastrointestinal and muscular symptoms. By demonstrating AgNPs’ ability to kill up to 59.53% of bradyzoites in contaminated tissue, our study suggests potential applications in meat processing or veterinary therapeutics, such as nanoparticle‐infused dips or feeds to disrupt transmission cycles. These findings also support the concept of integrating AgNPs into existing hygiene and food safety protocols, especially in endemic regions where conventional antiparasitic control is limited or ineffective. This addresses the need for eco‐friendly, non‐toxic alternatives to chemical antiparasitics, as AgNPs from green syntheses have shown reduced environmental impact and enhanced bioavailability. Furthermore, combining AgNPs with existing drugs, as seen in acetazolamide‐loaded variants against *Trichinella*, could enhance efficacy and minimize the development of resistance. Further investigations into synergistic combinations and optimized nanoparticle formulations are recommended to enhance therapeutic outcomes and minimize host cytotoxicity. Our work thus contributes to nanotechnology‐based strategies for parasitic control, potentially improving food safety in endemic regions (Abdel Hamed et al. 2023; Zhang et al. 2023). This finding fulfils the demand for eco‐friendly alternatives, with green‐synthesized AgNPs offering a lower environmental toll and better bioavailability.

The effective concentration of 800 µg/mL is relatively high, raising practical concerns regarding scalability, cost and potential cytotoxicity to mammalian cells, as well as the possibility of residues in meat products. Previous studies indicate that silver nanoparticles (AgNPs) can exhibit dose‐dependent cytotoxicity; however, green‐synthesized or surface‐modified variants frequently demonstrate improved biocompatibility at lower effective doses. To evaluate feasibility in meat processing or veterinary applications, further optimization is required, including reducing effective concentrations through synergistic combinations with other agents, targeted delivery systems or improved formulations, as well as comprehensive residue and safety assessments to ensure compliance with food safety regulations. In vivo pharmacokinetic and tissue distribution studies are also crucial before practical application can be considered (Frippiat et al. 2025; Noga et al. 2023). Overall, although the observed efficacy is limited, these findings establish foundational evidence for the use of AgNPs as a potential adjunct in integrated parasite control strategies, complementing broader efforts in nanotechnology for the treatment of parasitic diseases (AlGabbani 2023).

Despite these advancements, several limitations must be acknowledged. This study was conducted in vitro, which limits extrapolation to in vivo scenarios where host immune responses, pharmacokinetics and tissue penetration may alter outcomes. The relatively high concentrations required for peak efficacy (800 µg/mL) raise concerns about scalability and potential cytotoxicity to mammalian cells, although prior reports indicate low toxicity at therapeutic doses (Alexeree et al. 2024; Pescador‐Rojas et al. 2023). Moreover, particle aggregation and lack of standardized nanoparticle characterization may have contributed to variability in responses.

Uncharacterized nanoparticle variables, such as size and shape, may have influenced outcomes, as finer particles tend to enhance activity. Trypan blue, although dependable, overlooks sublethal metrics, such as metabolism, potentially underestimating its impact. Small samples inflated standard deviations, albeit with statistical import.

These constraints guide future endeavours. In vivo examinations in models such as sheep or cattle are crucial for evaluating efficacy, bioavailability and safety (AlGabbani 2023; Kaiaty et al. 2023). Green AgNPs or hybrids with selenium/copper oxide may enhance potency at reduced doses, as observed in nematodes (Kaiaty et al. 2023). Molecular and transcriptomic analyses could further elucidate the stress and apoptotic pathways triggered in bradyzoites following exposure to AgNPs. Gene expression analyses in treated bradyzoites could unravel resistance, with chronic host toxicity probes. Field trials in the meat sector would substantiate applications, tackling global parasitic zoonoses.

In essence, this in vitro probe confirms the dose‐ and time‐dependent antiparasitic effect of AgNPs on *Sarcocystis* bradyzoites, with mortality rates ranging from 20.11% at 100 µg/mL after 10 min to 59.53% at 800 µg/mL after 120 min. These findings demonstrate the potential of AgNPs as a supplementary antiparasitic strategy, particularly in contexts where conventional chemical drugs are less effective or have adverse environmental impacts. These underscore AgNPs’ promise against this parasite via membrane breach, ROS surge and intracellular havoc.

## 5 Conclusion

This work advances protozoan parasitology by providing preliminary evidence on AgNPs for *Sarcocystis*, addressing a long‐standing gap and laying the groundwork for nanotechnology interventions to counter traditional treatment resistance and enhance zoonotic oversight. However, future in vivo studies and biocompatibility assessments are warranted to validate these results and to ensure their safety for veterinary and food applications.

In closing, our efforts in spotlighting AgNPs in sustainable parasitology chart a path for safer, more effective tactics in veterinary care and food security, with extensive utility against rising parasitic threats.

## Author Contributions

Mir‐Hassan Moosavy and Nasser Hajipour conceived and designed the study. Nasrin Karimi conducted sampling and the experiments under the supervision of Mir‐Hassan Moosavy and Nasser Hajipour. Nasser Hajipour also performed the statistical analysis. Hamed Behniafar prepared the manuscript, whereas Mir‐Hassan Moosavy and Hamed Behniafar revised it. All authors reviewed and approved the final manuscript.

## Funding

The authors sincerely acknowledge the financial support provided by the Vice Chancellor for this MSc thesis at the University of Tabriz.

## Ethics Statement

All animal experiments were conducted in accordance with institutional ethical guidelines and were approved by the local ethics committee of the University of Tabriz.

## Conflicts of Interest

The authors declare no conflicts of interest.

## Data Availability

All relevant data are available from the corresponding authors and can be provided upon request.
